# A Proximity biotinylation assay with a host protein bait reveals multiple factors modulating enterovirus replication

**DOI:** 10.1371/journal.ppat.1010906

**Published:** 2022-10-28

**Authors:** Seyedehmahsa Moghimi, Ekaterina G. Viktorova, Samuel Gabaglio, Anna Zimina, Bogdan Budnik, Bridge G. Wynn, Elizabeth Sztul, George A. Belov

**Affiliations:** 1 Department of Veterinary Medicine and Virginia-Maryland College of Veterinary Medicine, University of Maryland, College Park, Maryland, United States of America; 2 Mass Spectrometry and Proteomics Resource Laboratory (MSPRL), FAS Division of Science, Harvard University, Cambridge, Massachusetts, United States of America; 3 Department of Cell, Developmental and Integrative Biology, University of Alabama at Birmingham; Birmingham, Alabama, United States of America; University of California, Irvine, UNITED STATES

## Abstract

As ultimate parasites, viruses depend on host factors for every step of their life cycle. On the other hand, cells evolved multiple mechanisms of detecting and interfering with viral replication. Yet, our understanding of the complex ensembles of pro- and anti-viral factors is very limited in virtually every virus-cell system. Here we investigated the proteins recruited to the replication organelles of poliovirus, a representative of the genus *Enterovirus* of the *Picornaviridae* family. We took advantage of a strict dependence of enterovirus replication on a host protein GBF1, and established a stable cell line expressing a truncated GBF1 fused to APEX2 peroxidase that effectively supported viral replication upon inhibition of the endogenous GBF1. This construct biotinylated multiple host and viral proteins on the replication organelles. Among the viral proteins, the polyprotein cleavage intermediates were overrepresented, suggesting that the GBF1 environment is linked to viral polyprotein processing. The proteomics characterization of biotinylated host proteins identified multiple proteins previously associated with enterovirus replication, as well as more than 200 new factors recruited to the replication organelles. RNA metabolism proteins, many of which normally localize in the nucleus, constituted the largest group, underscoring the massive release of nuclear factors into the cytoplasm of infected cells and their involvement in viral replication. Functional analysis of several newly identified proteins revealed both pro- and anti-viral factors, including a novel component of infection-induced stress granules. Depletion of these proteins similarly affected the replication of diverse enteroviruses indicating broad conservation of the replication mechanisms. Thus, our data significantly expand the knowledge of the composition of enterovirus replication organelles, provide new insights into viral replication, and offer a novel resource for identifying targets for anti-viral interventions.

## Author summary

Enteroviruses are arguably the most numerous group of viral pathogens infecting humans. Enterovirus infection can cause multiple complications including the development of asthma, type I diabetes, myocarditis, encephalitis, paralysis, and many others. Yet, there are no approved drugs against any of the pathogenic enteroviruses. The lack of effective anti-enterovirus measures is due in large part to insufficient understanding of molecular mechanisms supporting the development of enterovirus infections. Here, we developed a novel probe based on a host protein GBF1 that is universally required for enterovirus RNA replication to identify the proteins localized on enterovirus replication organelles, specialized membranous structures harboring the viral RNA replication complexes. Among the host proteins, we identified factors that promote and suppress the replication of diverse enteroviruses. Thus, our research provides new insights into molecular mechanisms of enterovirus replication and presents an important resource for the development of host-targeted anti-viral interventions.

## Introduction

Enterovirus infections are associated with numerous life-threatening and/or economically important diseases ranging from the common cold to fatal encephalitis. Among multiple pathogenic enteroviruses, licensed vaccines are available only against poliovirus and enterovirus A71, and there are no drugs approved by the FDA to control enterovirus infections [1–5]. This scarcity of effective anti-enterovirus measures reflects the inadequate understanding of the molecular mechanisms underlying the development of infections of this important group of viruses.

The *Enterovirus* genus belongs to the family *Picornaviridae* encompassing small positive-strand RNA viruses with non-enveloped icosahedral capsids that infect vertebrate hosts. Poliovirus is the best-studied enterovirus, its single genome RNA of about 7500 nucleotides is polyadenylated on the 3’-end and has a small protein VPg covalently attached to the 5’-end. The 5’-end long non-translated region of the genome RNA contains an internal ribosome entry site (IRES), and both 5’ and 3’ non-translated regions, as well as the coding part of the genome, contain *cis*-acting elements important for RNA replication [6]. The viral RNA is translated into a single polyprotein of about 200KDa which is processed by three viral proteases into three capsid and ten replication proteins, including stable intermediate products of the polyprotein processing [7–10]. The replication proteins form a replication complex where most of the viral proteins are assembled *in cis*, i. e. processed from the same polyprotein precursor, and likely initiate replication of the same RNA that served as an mRNA for protein synthesis [11–14]. The newly synthesized genomes can enter subsequent translation-replication cycles or be packaged into new viral particles.

The small genome size and consequently a limited repertoire of viral proteins implies that multiple host factors should support the replication process. Over the years, many host proteins that are required for, facilitate, or suppress the development of enterovirus infection have been identified (many of them are reviewed in [15–17]), but the full complement of all the cellular proteins involved in the enterovirus life cycle is yet to be defined. The two major groups that emerged from these studies are the host nucleic acid metabolism proteins that modulate translation and/or replication of the viral RNA, and membrane metabolism proteins that support the structural and functional development of viral replication organelles, specialized membranous platforms harboring the viral replication complexes. Currently, neither the stoichiometry of the viral proteins nor the full spectrum of the cellular factors required for the activity of the enterovirus replication complexes are known.

Multiple cellular factors can directly interact with specific structural elements of the viral RNA and affect its stability, translation, and replication efficiency. Importantly, while the enterovirus life cycle takes place exclusively in the cytoplasm, many of such proteins are normally restricted to the nucleus. Enteroviruses gain access to the nuclear proteins through the action of the protease 2A which specifically cleaves several nucleoporins resulting in the inactivation of organized nucleo-cytoplasmic trafficking and the release of nuclear proteins into the cytoplasm [18–21]. A few well-studied examples include a DNA repair component tyrosyl-DNA phosphodiesterase 2 (TDP2) which removes the VPg from the 5’ end of the viral RNA associated with polysomes [22,23]. Nuclear mRNA processing factors polypyrimidine tract binding protein 1 (PTB1) and poly(rC) binding proteins 1 and 2 (PCBP1 and PCBP2) interact with the IRES elements of different picornaviruses, including enteroviruses, and stimulate translation and/or replication of the viral RNAs [22,24,25]. Heterogeneous nuclear ribonucleoprotein C (HNRNPC) is required for optimal functioning of the poliovirus RNA replication complex [26]. Over the years, several other host nuclear proteins have been identified that interact with specific structural elements of the RNAs of enteroviruses and other picornaviruses, however, the full extent of the effects exerted by the complex mixture of the nuclear RNA-binding factors that are translocated to the cytoplasm upon infection is still poorly understood [15,27]. Interestingly, the accumulating evidence demonstrates that none of these RNA binding and/or processing proteins is absolutely required for enterovirus infection, suggesting a significant redundancy of the host protein functions in supporting viral RNA translation/replication cycle [22,23,28,29]. Rather, the cumulative effect of multiple host proteins on viral RNA stability, translation, and replication efficiency is likely cell type-dependent, and may contribute to the determination of viral tropism in the host [30,31].

Viral RNA replication and, likely, translation, especially during the later stages of infection, are associated with replication organelles. Thus, the replication organelles are expected to be significantly enriched in host factors that support the translation and/or replication of the viral RNA, but also those that may possess a direct anti-viral activity. These structures feature unique membrane and protein composition, and their establishment and expansion depend on virus-induced reconfiguration of the cellular lipid and membrane synthesis and trafficking pathways [32,33]. Accordingly, several key host proteins hijacked by enteroviruses have been shown to mediate the structural development and the acquisition of a specific replication-conducive biochemical signature of the replication organelles. CTP-phosphocholine-cytidyl transferase alpha (CCTα), the rate-limiting enzyme in the phosphatidylcholine synthesis pathway, lipid droplet-associated lipases adipocyte triglyceride lipase (ATGL) and hormone-sensitive lipase (HSL), as well as several long-chain acyl-CoA synthetases (ACSLs), are implicated in the activation of infection-specific phospholipid synthesis that provides the bulk of membrane material for the expansion of the replication organelles [34–37]. Recruitment of GBF1, a guanine nucleotide exchange factor for small Arf GTPases (ArfGEF), results in a massive association of Arfs with the replication organelles. Phosphatidylinositol 4 kinase III beta (PI4KIIIβ) and oxysterol binding protein (OSBP) together with several other factors mediate the enrichment of the replication organelles in phosphatidylinositol 4 phosphate (PI4P) and cholesterol. Interestingly, two distinct pathways of recruitment of PI4KIIIβ to and accumulating of PI4P on the replication organelles have been identified. One depends on the engagamnent of a cellular protein ACBD3 by the viral protein 3A, while the other relies on the viral protein 3CD and is dependent on GBF1 and Arf activation [38,39]. Inhibition of GBF1, PI4KIIIβ or OSBP activities severely restricts the replication of diverse enteroviruses [39–53].

Here we took advantage of a strict dependence of enterovirus replication on GBF1 to perform a proteomics characterization of the replication organelles. GBF1 is recruited to the replication organelles through direct interaction with the enterovirus non-structural protein 3A, and the ArfGEF activity of GBF1 is required to support viral RNA replication [50–52]. Thus, GBF1 localizes on the replication organelles close to the active replication complexes and can be used to probe the local micro-environment. GBF1 is a large multi-domain protein normally engaged in multiple protein-protein and protein-membrane interactions (reviewed in [54]). We previously demonstrated that the C-terminal part of GBF1 is dispensable for enterovirus replication [55,56]. To reduce interactions of GBF1 with proteins not relevant for viral replication, we used a C-terminally truncated GBF1 to generate a fusion with the APEX2 peroxidase. This peroxidase in the presence of H_2_O_2_ and biotin-phenol generates short-lived active biotin-phenoxyl radicals that covalently attach to electron-rich amino-acids of nearby proteins. The APEX2-based proximity biotinylation assay has been successfully used to characterize the proteomes of different compartments of eukaryotic cells [57–59]. In non-infected cells, the truncated APEX2-GBF1 construct is diffusely localized in the cytoplasm, but in infected cells, it was effectively recruited to the replication organelles and was fully functional in supporting poliovirus replication. Accordingly, the profile of the biotinylated proteins isolated from mock- and poliovirus-infected cells was significantly different. Among the biotinylated viral proteins, i.e. those localized close to GBF1, the intermediate products of polyprotein processing were significantly enriched, suggesting that GBF1 environment is associated with active polyprotein processing, and/or that those incomplete products of proteolysis may perform specific functions in the GBF1-enriched domains of the replication organelles. The largest group of host proteins identified in infected cells were those involved in RNA metabolism, many of which are normally localized in the nucleus. This underscores the massive relocalization of nuclear proteins upon infection and their engagement in the replication process. Many of these proteins have been previously reported to be associated with the replication of diverse enteroviruses, validating our experimental approach. Knock-down of expression of several of the most abundant proteins identified in our assay revealed both pro- and anti-viral factors, affecting translation/replication step of the viral RNA life cycle. Interestingly, one of the strongest negative effects on viral replication was observed upon the knock-down of expression of fructose-bisphosphate aldolase A (AldoA), a glycolytic enzyme important for ATP biogenesis and the production of ribose-5-phosphate, a substrate for *de novo* nucleotide synthesis. We observed similar pro- and anti-viral effects of the depletion of the assayed proteins on the development of infection of poliovirus and Coxsackie virus B3, representatives of the Enterovirus C and B species, respectively, indicating a conservation of the enterovirus replication mechanisms.

Together, our findings significantly expand the repertoire of known cellular proteins involved in the development of enterovirus infection and elucidate the complex composition of pro- and anti- viral factors associated with the replication organelles.

## Results

### Establishment and characterization of an APEX2-GBF1 system for proximity biotinylation

**Fig 1:**
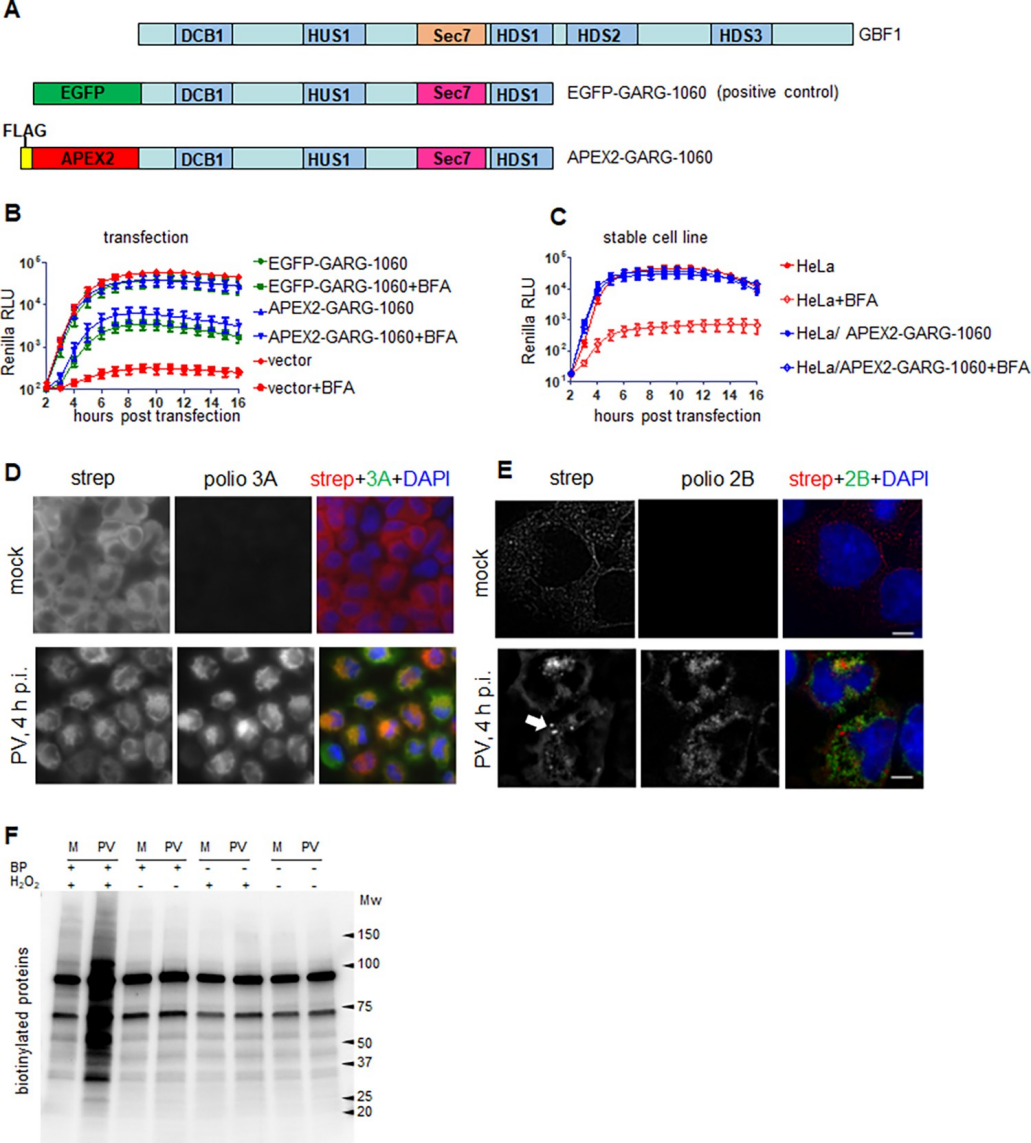
Characterization of the APEX2-GARG-1060 proximity biotinylation system. **A.** Domain organization of GBF1 and the C-terminally truncated GBF1 constructs fused to EGFP (positive control) and APEX2. Both GBF1 truncated constructs contain the BFA-resistant Sec7 domain from ARNO. **B.** Polio replicon replication was assessed in the presence or absence of 2μg/ml of BFA in HeLa cells transfected with plasmids expressing the C-terminally truncated GBF1 fusions with APEX2 or EGFP, or an empty vector **C.** Polio replicon replication assay was performed in control HeLa cells, or HeLa cells stably expressing APEX2-GARG-1060 with or without 2μg/ml of BFA. **D.** HeLa cells stably expressing APEX2-GARG-1060 were infected (or mock-infected) with 10 PFU/cell of poliovirus, and the biotinylation reaction was performed at 4 h p.i. The cells were processed for visualization of biotinylated proteins with a fluorescent streptavidin conjugate and immunostaining for a poliovirus antigen 3A. **E.** HeLa cells stably expressing APEX2-GARG-1060 were infected (or mock-infected) with poliovirus and the biotinylation reaction was performed as in D. The cells were stained with a fluorescent streptavidin conjugate and antibodies against a poliovirus antigen 2B and processed for structural illumination superresolution (SIM) microscopy. The arrow shows biotinylation-positive structures identified as stress granules. The scale bar is 10μm. **F.** HeLa cells stably expressing APEX2-GARG-1060 were infected (PV), or mock-infected (M) with 10 PFU/cell of poliovirus, and protein biotinylation was assessed after performing the biotinylation reaction at 4 h p.i. with biotin-phenol (BP) and hydrogen peroxide (complete reaction), or without one, or both compounds.

The Arf-activating function of GBF1 and other ArfGEFs is mediated by their catalytic Sec7 domains. The fungal metabolite brefeldin A (BFA) inhibits the Sec7 function of GBF1 and some other but not all ArfGEFs [60]. Previously, we generated a GBF1 construct containing a Sec7 domain from another cellular ArfGEF, ARNO, which is not sensitive to BFA (GARG, from GBF1-ARNO-GBF1) [61]. The advantage of such BFA-insensitive constructs is that it is possible to explore the GBF1-related functions supported exclusively by the exogenously introduced BFA-insensitive GBF1 derivatives after inactivating the endogenous GBF1 by BFA. We also previously established that the C-terminal part of GBF1 downstream of the HDS1 domain is dispensable for viral replication [55,56]. We reasoned that for probing the proteome of replication organelles such a “minimal” GBF1 construct lacking the C-terminus would be advantageous since all the interactions of the C-terminal part of GBF1 that are non-essential for viral replication will be eliminated. Thus, we fused APEX2 peroxidase N-terminally to the GARG truncated at the end of the HDS1 domain at amino acid 1060 (APEX2-GARG-1060 construct, Fig 1A). We also introduced a FLAG epitope at the very N-terminus of the construct. To assess the ability of the APEX2-GARG-1060 to function in viral replication, HeLa cells were transfected with a plasmid expressing APEX2-GARG-1060, a plasmid expressing EGFP-GARG-1060 (positive control), or an empty vector (negative control). The next day, the cells were transfected with a polio replicon RNA expressing a *Renilla* luciferase gene instead of the capsid proteins, and replication was monitored in the presence and in the absence of BFA. The inhibitor blocked replication in cells transfected with an empty vector, but cells expressing either APEX2- or EGFP-GARG fusions similarly supported replication in the presence of the drug (Fig 1B). Thus, the APEX2-GARG-1060 construct is fully functional in polio replication.

Transiently-transfected cells are not well suited for proteomics studies because the level of transgene expression varies greatly and also because the transfection reagents and presence of a plasmid DNA in the cytoplasm could trigger innate immune responses. Thus, we established a stable cell line expressing the APEX2-GARG-1060 construct by a retrovirus vector transduction followed by clonal selection so that the resulting culture expressed a uniform level of the transgene. The polio replicon replicated equally efficiently in these cells in the presence and in the absence of BFA, while in control HeLa cells BFA blocked the replicon replication (Fig 1C). This cell line was used for all further experiments, all of which were performed in the presence of BFA so that the replication was supported exclusively by the APEX2-GARG-1060 construct.

To assess APEX2-GARG-1060-mediated protein biotinylation, the cells were infected (or mock-infected) with poliovirus at a multiplicity of infection (MOI) of 10 plaque-forming units (PFU)/cell (so that all cells are infected) and the biotinylation reaction was performed at 4 hours post-infection (h p.i.), which corresponds to the middle of poliovirus replication cycle. The cells were then stained with a fluorescent streptavidin conjugate to visualize the biotinylated proteins, and with an antibody against a viral non-structural antigen 3A. In mock-infected cells, the streptavidin signal was diffusely distributed in the cytoplasm (although higher magnification images showed some staining of the intracellular structures), consistent with the loss of GBF1-specific subcellular localization of the APEX2-GARG-1060 construct due to the removal of the C-terminal part of GBF1, containing most of the Golgi membrane-targeting determinants [62,63] (Fig 1D, mock). In infected cells, however, the biotinylation pattern was strictly confined in bright perinuclear rings, the characteristic localization of poliovirus replication organelles, and, accordingly, was extensively co-localized with the 3A signal (Fig 1D, PV). It was also visibly brighter than the streptavidin signal in mock-infected cells. We also investigated the fine distribution of biotinylated proteins using structural illuminated superresolutiuon microscopy (SIM). The SIM images showed a reticular pattern in mock-infected cells that likely represents the mitochondria which are enriched in biotin-containing enzymes [64]. In contrast, in infected cells, a differently structured biotinylation signal was associated with the replication organelles, as evidenced by the staining for the viral replication antigen 2B (Fig 1E). Interestingly, we observed in many infected cells bright round foci of biotinylation signal (Fig 1E, arrow) which were identified as stress granules (see the last section in the Results).

To characterize the specificity of the biotinylation reaction, cells were similarly infected (or mock-infected), and at 4 h p.i. incubated either with both H_2_O_2_ and biotin-phenol, or with H_2_O_2_ or biotin-phenol only, or without any of those compounds. Western blot analysis with streptavidin conjugated to horseradish peroxidase (HRP) showed two major bands of similar intensity in all the samples, likely corresponding to endogenous biotin-containing enzymes previously observed in studies utilizing APEX2-mediated biotinylation [64]. Importantly, only cells incubated with both compounds showed extensive biotinylation of additional proteins, confirming the specificity of the biotinylation reaction (Fig 1F). The level of protein biotinylation in infected cells significantly exceeded that in the mock-infected sample, in accordance with the pattern observed after staining of cells with a fluorescent streptavidin conjugate.

Thus, APEX2-GARG-1060 efficiently supports poliovirus replication, is recruited to replication organelles and can specifically biotinylate proteins associated with these structures.

### Initial characterization of biotinylated proteins during the time course of infection

#### Cellular proteins

**Fig 2:**
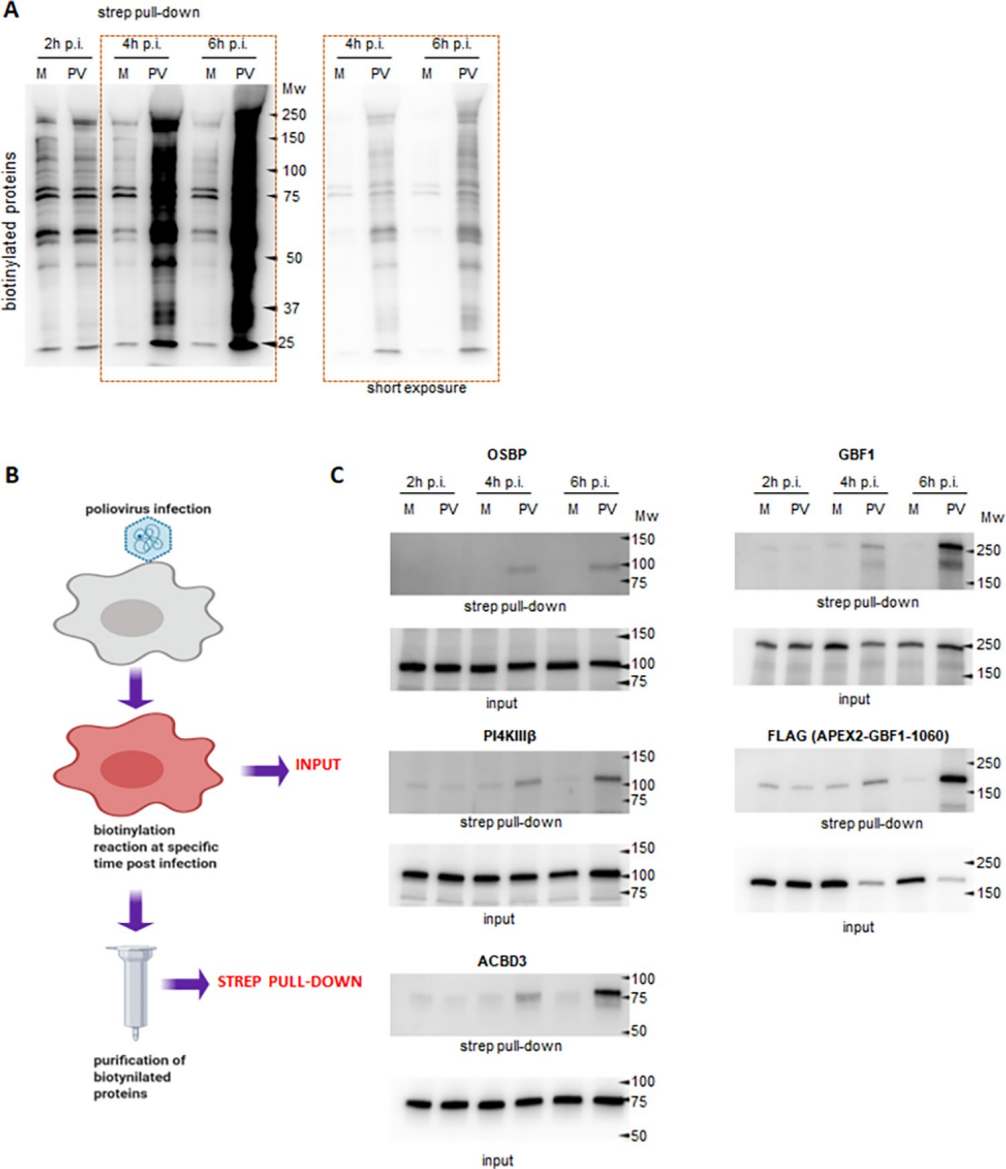
Known cellular proteins recruited to replication organelles are biotinylated by FLAG-APEX2-GARG1060. **A.** HeLa cells stably expressing FLAG-APEX2-GARG-1060 were infected (PV), or mock-infected (M) with 10 PFU/cell of poliovirus, and biotinylation was performed at the indicated times post-infection. Biotinylated proteins were collected on streptavidin beads, resolved on SDS-PAGE and analyzed in a Western blot with HRP-conjugated streptavidin. **B.** Scheme of the biotinylation experiment to compare the biotinylated proteins (strep pull-down) with the proteins in the lysates before fractionation (input). The figure was made using Biorender templates. **C.** HeLa cells stably expressing APEX2-GARG-1060 were infected (PV), or mock-infected (M) with 10 PFU/cell of poliovirus, the biotinylation reactions were performed at 2, 4, and 6 h p.i., and the unfractionated cellular lysates (input) and the isolated biotinylated proteins (strep pull-down) were analyzed in a western blot with the indicated antibodies. Anti-FLAG antibodies detect the APEX2-GARG-1060 protein.

An important advantage of the APEX2-based biotinylation is the short time of the labeling reaction that permits time-resolved snapshots of protein composition. The replication cycle of poliovirus in HeLa cells takes about 6–8 h. We infected cells expressing APEX2-GARG-1060 with 10 PFU/cell of poliovirus and performed the biotinylation reactions at 2, 4 and 6 h p.i. The biotinylated proteins were isolated on streptavidin beads and analyzed in a western blot-type assay with a streptavidin-HRP conjugate to obtain the global biotinylation pattern. At 2 h p.i., the amount of biotinylated proteins and their pattern were similar in infected and control samples, and the mock-infected samples did not significantly change during the time course of the experiment. At 4 and 6 h p.i., protein biotinylation strongly increased in infected cells, and they were distributed throughout a wide range of molecular weights. Visually, the pattern of the biotinylated proteins from the 6 h p.i. sample was the same as at 4 h p.i., but the amount of the biotinylated proteins was higher (Fig 2A).

We then analyzed if the cellular factors ACBD3, OSBP, and PI4KIIIβ known to be involved in PI4P and cholesterol enrichment of the replication organelles [39,40,47,48] are present in the biotinylated fraction, suggestive of close association with GBF1 in infected cells. The infection and biotinylation reaction were performed as for Fig 2A. The unfractionated lysates and the proteins recovered in the biotinylated fraction were analysed in western blot with the indicated antibodies (Fig 2B). We observed a specific increase of ACBD3, OSBP, and PI4KIIIβ signals in the biotinylated fraction only in the infected cells collected at 4, and especially at 6 h p.i. Biotinylated OSBP signal was always observed only in infected cells, while traces of PI4KIIIβ and ACBD3 were also visible in the biotinylated material recovered from mock-infected cells (Fig 2C). We also analyzed the biotinylation of endogenous GBF1, and the APEX2-GARG-1060 construct itself. Since the APEX2-GARG-1060 construct lacks the C-terminal part containing the epitope recognized by the anti-GBF1 antibody, it was detected with anti-FLAG antibodies. Again, the strongest signals for both biotinylated GBF1 and APEX2-GARG-1060 were observed in cells infected for 4 and 6 h (Fig 2C). The increase of APEX2-GARG-1060 signal in 4 and 6 h p.i. samples coincided with a noticeable decrease of the corresponding signal in the total input material, likely reflecting the degradation of the cytoplasmic, but not replication-organelle associated pools of this protein (Fig 2C). Surprisingly, we did not detect biotinylated Arfs, even though they are enriched on the replication organelles [65,66], and at least a fraction of Arf molecules is expected to be localized close to GBF1.

#### Viral proteins

**Fig 3:**
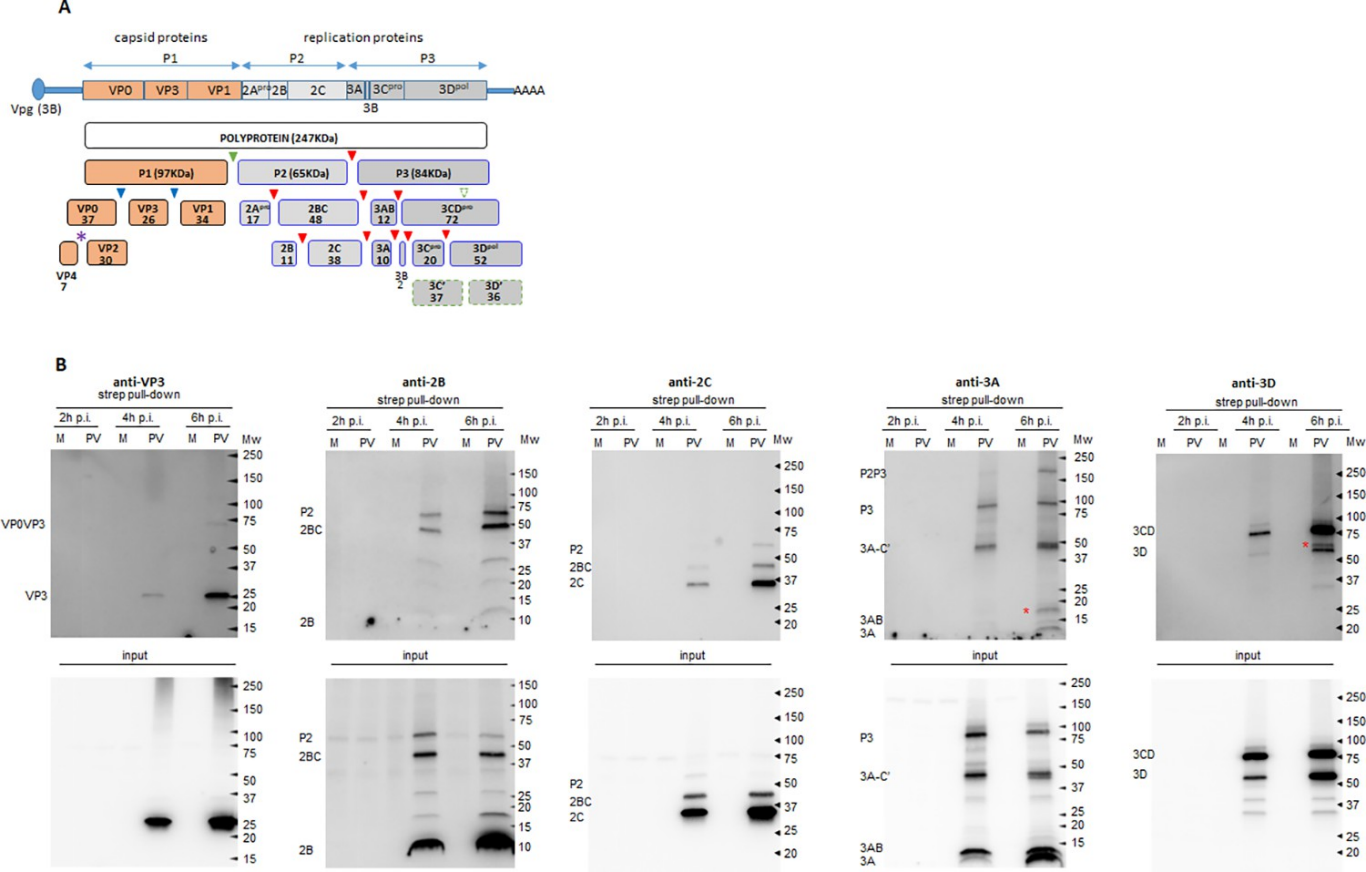
Biotinylation of viral proteins by APEX2-GARG-1060. **A.** Poliovirus genome and polyprotein processing scheme. The cleavage sites for the viral proteases 2A, 3C, and 3CD are indicated by green, red, and blue-filled triangles, respectively. The dashed empty green triangle indicates a 2A cleavage site in 3D believed to be dispensable for replication. The purple star indicates the autocatalytic cleavage site in VP0. **B.** HeLa cells stably expressing APEX2-GARG-1060 were infected (PV), or mock-infected (M) with 10 PFU/cell of poliovirus, and biotinylation reactions were performed at the indicated times post-infection. Unfractionated cellular lysates (input) and isolated biotinylated proteins were analyzed in a Western blot with indicated antibodies. The antibodies recognize the final and intermediate polyprotein cleavage products containing the corresponding antigen. Red stars on anti-3A and anti-3D panels indicate polyprotein fragments that do not match the known polyprotein cleavage products.

The proximity biotinylation approach allowed us to identify specific fragments of the poliovirus polyprotein localized in the vicinity of APEX2-GARG-1060 on the replication organelles. Since the poliovirus genome is expressed as a single polyprotein undergoing a proteolytic processing cascade, antibodies against a given antigen will recognize the final maturation product and all the intermediate cleavage products containing this antigen. The available panel of antibodies suitable for western blot (VP3, 2B, 2C, 3A, 3D) covers all known intermediate fragments of the poliovirus polyprotein processing and all individual proteins except capsid proteins VP0 and VP1, proteases 2A and 3C, and the RNA replication protein primer 3B (VPg) (Fig 3A). As shown in Fig 3B, all the tested viral antigens were present in the biotinylated fraction. Interestingly, while in the input material the final polyprotein cleavage products were most abundant, in the biotinylated protein fraction the intermediate polyprotein cleavage products were overrepresented. For example, the 6 h p.i input material analysed with anti-3A antibody shows the major signals in the fully-processed 3A and 3AB proteins (low molecular weight section, below 15KDa). Yet, in the biotinylated fraction, signals for 3A and 3AB, while clearly detectable, are weaker than the uncleaved precursors P3 and P2P3 (~90 and ~150 KDa, respectively) (Fig 3B, anti-3A panel). Similarly, in the samples analyzed with anti-2B antibody, in the input, the fully processed 2B (~10KDa) represents the major signal, while in the biotinylated fraction, 2B is also detected, but the major 2B signal is found in the intermediate cleavage products 2BC and P2 (~50 and ~65 KDa, respectively) (Fig 3B, anti-2B panel). We also observed a specific increase in the biotinylated fraction of the viral antigen-positive fragments that do not correspond to the canonical products of the viral polyprotein processing. Note the red asterisks marking a 3A-positive fragment in the 15-20KDa range (Fig 3B, anti-3A panel, 6 h p.i), or a 3D-positive fragment of a molecular weight slightly higher than 3D (Fig 3B, anti-3D panel, 6 h p.i.). This may suggest that the APEX2-GARG-1060 (and therefore, *bona fide* GBF1) environment is associated with active polyprotein maturation.

Collectively, these results demonstrate that APEX2-GARG-1060 in infected cells can specifically biotinylate both viral and host proteins associated with replication organelles and that 6 h p.i. samples are the most representative for the characterization of the proteome of the replication organelles.

### Proteomics characterization of the replication organelles

**Fig 4:**
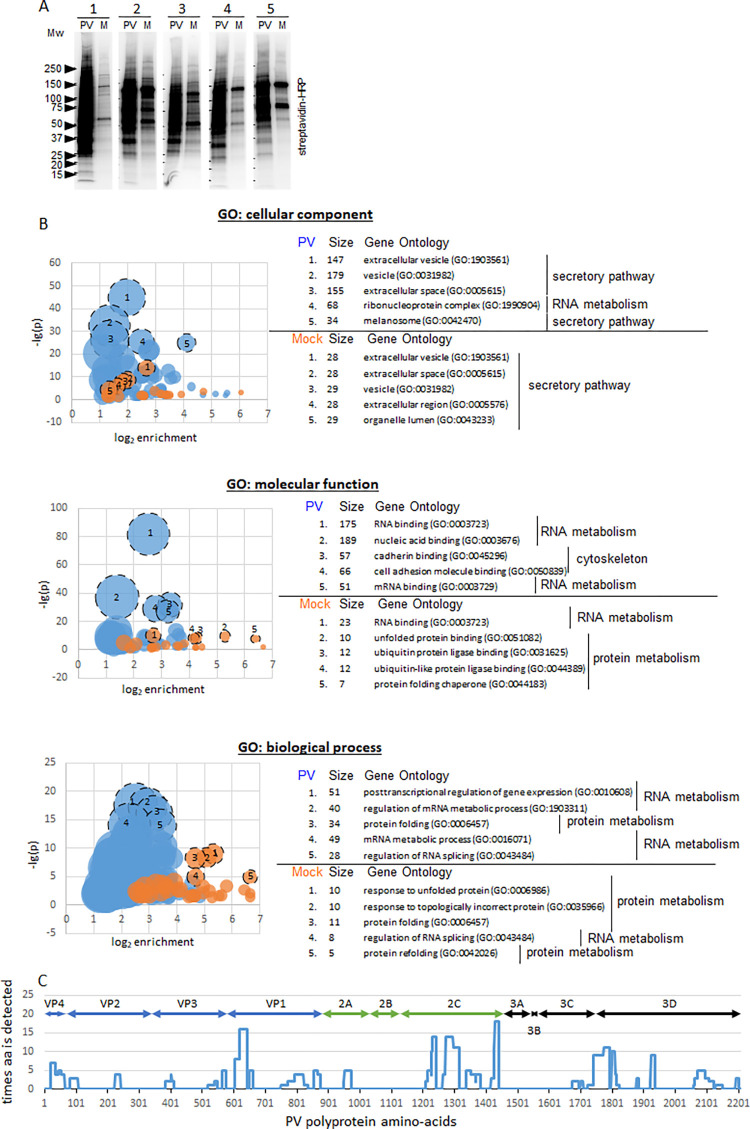
Characterization of the proteome biotinylated by FLAG-APEX2-GARG1060. **A.** In five independent experiments, HeLa cells stably expressing APEX2-GARG-1060 were infected (PV), or mock-infected (M) with 10 PFU/cell of poliovirus, and biotinylation was performed at 6 h p.i. Biotinylated proteins were purified on streptavidin beads and analyzed in a Western blot with HRP-streptavidin. Biotinylated proteins from these five experiments were pooled for proteomics analysis. Full proteomics data are available in S1 Data. **B.** Gene ontology (GO) enrichment analysis of the proteomics data using PANTHER classification system [69]. Buble graphs show the number of proteins associated with a particular GO term (bubble size), the log_2_ of enrichment over the expected non-specific associations of genes in the dataset with a particular GO term (x-axis), and the statistical significance of the observed enrichment (negative log_10_ of p-value, y-axis). Five of the most statistically significantly enriched GO terms for proteins from infected and mock-infected samples are shown. The full GO analysis is available in S2 Data. **C.** The distribution of the poliovirus-specific peptides identified by mass-spectrometry analysis across the poliovirus polyprotein. The x-axis shows amino-acid positions in the poliovirus polyprotein, the y-axis shows how many times a particular amino-acid was detected.

For the proteomics analysis, HeLa cells stably expressing APEX2-GARG-1060 were grown in 225 cm^2^ flasks and infected (or mock-infected) with poliovirus at an MOI of 10, the biotinylation reaction was performed at 6 h p.i. for 3 min, and the biotinylated proteins were purified on streptavidin beads. Five independent experiments were performed, and the aliquots of the isolated proteins were assessed in a western blot assay with a streptavidin-HRP conjugate. In all experiments, a similar pattern of a highly increased amount of biotinylated proteins in infected cells was observed, as expected (Fig 4A). The rest of the purified biotinylated proteins from each experiment were pooled together and processed for mass-spectrometry protein identification and label-free quantitation (LFQ). Upon filtering the identified proteins from common contaminants, as well as from carboxylases which contain naturally covalently attached biotin, and proteins with peroxidase activity which can likely be biotinylated independently of APEX2, 369 and 43 proteins were enriched in the infected and non-infected sets, respectively. 192 proteins in the infected sample and 37 proteins in the mock-infected sample were identified from 2 or more peptides. 331 proteins were found exclusively in the infected sample, while just 5 proteins from the mock-infected sample were not detected in the infected sample (S1 Data). Among the cellular proteins detected in the biotinylated pool by our western blot analysis (see Fig 2), the proteomic analysis identified GBF1 (Q92538) from a total of nine peptides, five of them unique (9 total:5 unique; further on this designation is used for peptides detected for each protein), while ACBD3 (Q9H3P7) and OSBP1 (P22059) proteins were identified from one peptide each, and PI4KIIIβ (Q9UBF8) was not detected (S1 Data).

Since the proteins were biotinylated by a GBF1-based construct, we expected the presence of at least some known interactors of GBF1 or Arf GTPases. Indeed, analysis of the identified proteins using the Biogrid database of curated interaction data [67,68] identified 44 GBF1 interactors, 34 of which were found only in the infected sample (S3 Data). Moreover, 17 Arf1, one Arf3, four Arf4, three Arf5, and four Arf6 interactors were identified in the biotinylated pool (S3 Data). All Arf3, Arf4 and Arf5 interactors, as well as 12 Arf1 and two Arf6 interactors were identified exclusively among the proteins from the infected sample.

The global association of the biotinylated proteins with cellular structures and pathways was analyzed by Gene Ontology (GO) term enrichment in the cellular component, molecular function, and the biological process categories using the PANTHER classification system [69]. In general, the GO term enrichment of proteins from the infected sample had a much higher statistical significance than those from the mock-infected cells, consistent with the difference in the number of proteins in each sample. Across both samples, the most statistically significantly enriched categories included proteins associated with the cellular secretory pathway and chaperone-assisted protein folding. In the mock-infected sample, proteins associated with the proteasome-dependent protein degradation were among the highly enriched. In the infected sample, a significant amount of proteins were also associated with the cytoskeleton. Yet, the nucleic acid, and in particular RNA metabolism, emerged as the predominantly enriched GO terms from the infected sample (Fig 4B and S2 Data). Interestingly, 17 proteins were associated with dsRNA binding, 10 of which were present only in the infected sample (S3 Data).

A literature search revealed that 62 of the biotinylated proteins identified in the infected sample were previously reported to have a functional significance for the replication of different enteroviruses, while 50 more were detected in high-throughput screens as proteins that undergo some changes upon enterovirus infection, or as interacting partners of the viral proteins, but their functional significance has not been investigated (S4 Data). The known constituents of the poliovirus replication complex, KH domain-containing, RNA-binding, signal transduction-associated protein 1 (KHDRBS1, Sam68) [70] was identified from 8 total: 2 unique peptides; splicing factor, proline- and glutamine-rich (SFPQ) [71] from 10 total: 6 unique peptides, and polyadenylate-binding protein 1 (PABCP1) [72] from 3 total: 2 unique peptides, exclusively in the infected sample. 6 total:3 unique peptides shared between poly-(rC)-binding proteins 1 and 2 (PCBP1, 2) [73–75] were identified in the infected sample while one unique peptide for each PCBP1 and PCBP2 was detected in the mock-infected control. Polypyrimidine tract-binding protein 1 (PTB1) involved in the activation of the enterovirus IRES-driven translation [76,77] was detected by one peptide in both infected and mock-infected samples (S1 Data).

The poliovirus-specific peptides (207 total: 64 unique) identified by mass-spectrometry analysis were distributed along the whole viral polyprotein, with an intriguing absence of peptides covering the 2B and 3A-3B regions. An increased clustering of the detected peptides was observed in the N-terminus of a capsid protein VP1, 3C-3D junction, and particularly in the 2C region (Fig 4C). This pattern is in accordance with the data shown in Fig 3B indicating that the APEX2-GARG-1060 environment on the replication organelles is enriched in all viral structural and replication proteins.

Overall, these data validate the relevance of the identified cellular proteins for poliovirus replication and provide an important insight into the viral and cellular protein environment of the replication organelles in the proximity of GBF1.

### Identification of novel host factors affecting viral replication

One of the major goals of this study was to identify novel cellular factors important for viral replication. The selection of proteins from a large proteomics dataset for further analysis is inevitably arbitrary, but our general criteria were that the proteins should be detected from multiple peptides in the infected sample, not have a previously characterized role in enterovirus replication, and represent different functional clusters.

We selected the following groups for analysis: Group 1) Glycolytic enzymes: fructose-bisphosphate aldolase A (AldoA; detected from 32 total:15 unique peptides), pyruvate kinase M (PKM; detected from 15 total:11 unique peptides), and L-lactate dehydrogenase chain A and B (LDHA and LDHB; detected from 6 total:2 unique and 4 total: 2 unique peptides, respectively). Apart from being highly enriched, this group of glycolytic enzymes was selected because LDHA and LDHB are reported Arf interactors and because the glycolytic pathway provides substrates for *de novo* nucleotide synthesis, which may be important for rapidly replicating RNA viruses [67,68,78–80].

Group 2) RNA binding proteins: Heterogeneous nuclear ribonucleoproteins A0, H2, H3, R, U (HNRNPA0, H2, H3, R, U; detected from 10:3, 15:6, 8:5, 10:6, 27:11 of total:unique peptides, respectively), heterogeneous nuclear ribonucleoprotein Q (SYNCRIP; detected from 8 total:6unique peptides), Ewing Sarcoma Breakpoint Region 1 (EWSR1; detected from 12 total:5 unique peptides), and RNA-binding motif protein, X chromosome (RBMX; detected from 7 total:6 unique peptides) were among the most enriched in the infected sample. SYNCRIP and HNRNPU were previously reported in a proteomics screen detecting proteins bound to poliovirus RNA. The depletion of HNRNPU did not affect the virus yield, the contribution of SYNCRIP was not analyzed [81].

Group 3) Potential antiviral factors: A dsRNA binding protein ILF3 was identified from 8 total:4 unique peptides in the infected sample. ILF3 was shown to be important for the establishment of dsRNA-induced anti-viral signaling and to either promote or inhibit the replication of diverse viruses [82–86]. The ILF3 gene is expressed as multiple isoforms of the two major variants of 90KDa and 110KDa proteins. Both 90KDa and 110KDa proteins have two dsRNA binding domains, with an extended C-terminal GQSY-rich region in the latter [87]. A 90KDa isoform of ILF3 was demonstrated to inhibit translation of a poliovirus-rhinovirus chimera RNA in a cell-type dependent manner by binding to the rhino- but not poliovirus IRES [88].

We screened the effects of siRNA-mediated depletion of these proteins on polio replicon replication and on the accumulation of the viral protein 2C upon infection. In the replicon assay, the RNA is transfected into the cells, bypassing the normal virion-receptor mediated delivery, so it reflects the RNA translation-replication step of the viral life cycle, while the accumulation of 2C upon infection also integrates the effects of virion-receptor interaction, penetration and uncoating. As a control for our siRNA-based screening analysis, we also included siRNA against KHDRBS1 (Sam68) (identified from 8 total: 2 unique peptides in our proteomics dataset), which was previously shown to bind poliovirus polymerase 3D and promote viral replication [70].

Depletion of Sam68 inhibited polio replication in both replication and infection assays, as expected, thus validating our approach (S1 Fig). Among all the proteins we tested, only depletion of LDHA was toxic to cells, making its specific effect on polio replication impossible to evaluate in this system. Depletion of PKM, LDHB, SYNCRYP and HNRNPU affected the replication in only one of the assays, suggesting a moderate contribution of these proteins in the virus life cycle, at least under cell culture conditions. Curiously, depletion of RBMX strongly increased the replicon replication but decreased the accumulation of 2C upon infection (S1 Fig). Depletion of AldoA, HNRPA0 and EWSR1 showed consistent negative effects on polio replication in both assays, while depletion of HNRNPR, HNRNPH2, HNRNPH3, and in particular the 90KDa isoform of ILF3 strongly increased replication in both assays (S1 Fig).

Thus, our analysis reveals novel host factors with stimulatory and inhibitory effects on poliovirus replication.

### AldoA, EWSR1 and ILF3-90 similarly control the replication of diverse enteroviruses

**Fig 5:**
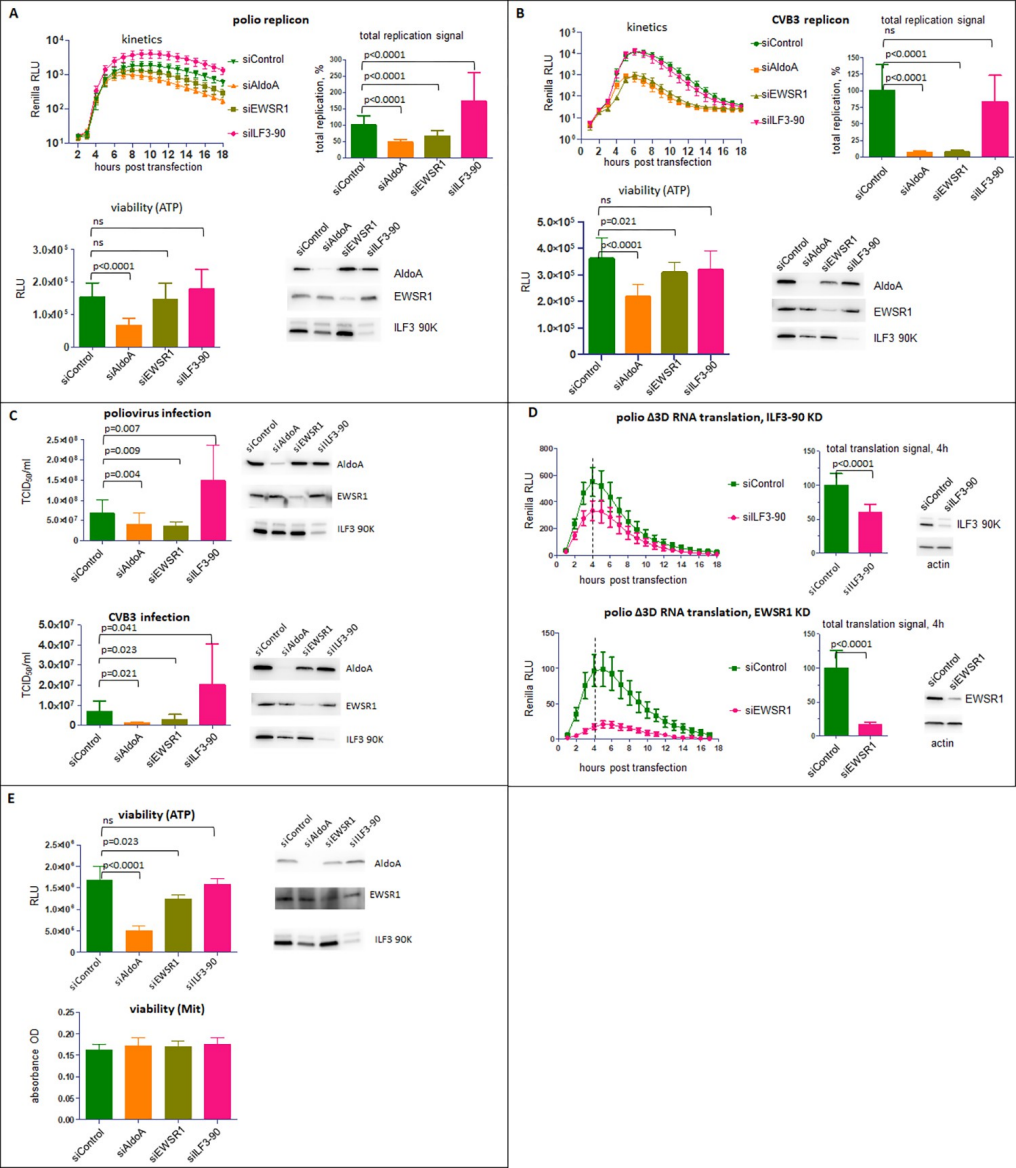
Effects of siRNA-mediated knockdown of expression of AldoA, EWSR1 and ILF3-90 on enterovirus replication. **A, B.** HeLa cells were transfected with siRNAs specific to AldoA, EWSR1 and 90KDa isoform of ILF3, or non-targeting control siRNA, and polio or Coxsakie B3 replicon replication assays were performed 72 h post siRNA transfection. The total replication signal was calculated as the area under the corresponding kinetics curves. Cell viability signal is proportional to the level of ATP in cells. Western blots show the efficacy of siRNA-mediated knockdown of the targeted proteins. **C.** HeLa cells were transfected with siRNAs specific to AldoA, EWSR1 and 90KDa isoform of ILF3, or non-targeting control siRNA. 72 h post siRNA transfection cells were infected with an MOI of 1 PFU/cell of poliovirus or Coxsackie virus B3, and the total virus yield was determined at 6 h p. i. Western blots show the efficacy of siRNA-mediated knockdown of the targeted proteins. **D.** HeLa cells were transfected with siRNAs specific to 90KDa isoform of ILF3 or EWSR1 or a non-targeting control siRNA. 72h post siRNA transfection, the cells were transfected with a replication-defective replicon RNA containing the Δ3D mutation. The total translation signal was calculated as the area ander the curve from 1 to 4 h post Δ3D RNA transfection. Western blots show the efficacy of siRNA-mediated knockdown of the targeted proteins. **E.** HeLa cells were transfected with siRNAs specific to AldoA, EWSR1 and 90KDa isoform of ILF3, or non-targeting control siRNA, and cell viability assays detecting the level of ATP or the activity of the mitochondrial respiratory chain enzymes were performed 72h post siRNA transfection. Western blots show the efficacy of siRNA-mediated knockdown of the targeted proteins.

The proteins AldoA, EWSR1, and the 90K isoform of ILF3 whose depletion consistently affected polio replication were selected for a more detailed analysis. The siRNA knockdown of AldoA and EWSR1 expression significantly inhibited the replication of both polio and Coxsackie B3 replicons. For polio replicon, the total replication signal was reduced to ~45% and 65% of control in AldoA and EWSR1- depleted cells, respectively, and for Coxsackie B3 replicon the replication signal reached less than 10% of that of control in both cases. The effect of ILF3-90 depletion was seen only with the polio replicon, with the increase of the total replication signal to ~ 150% of control (Fig 5A and 5B). In conditions of *bona fide* virus infection, the depletion of AldoA and EWSR1 inhibited, while the depletion of ILF3-90 stimulated the infectious virion yield of both viruses (Fig 5C). The AldoA and ESWR1 depletions, similarly to the replicon replication assay, affected Coxsackie B3 virus more than poliovirus with the statistically significant decrease of virus productions down to ~50–60% for poliovirus and ~16–20% for Coxsakievirus B3, relative to the respective controls. The depletion of ILF3-90 increased the production of poliovirus to ~200% and that of Coxsackie B3 virus to ~300% relative to controls (Fig 5C).

EWSR1 and ILF3-90 are RNA-binding proteins and may directly affect the RNA translation and the RNA replication steps of the enterovirus life cycle. During infection, both processes are interdependent and cannot be studied separately. To determine the effect of the EWSR1 and ILF3-90 depletion on the translation step only, we transfected cells with a polio replicon RNA expressing Renilla luciferase with a deletion in the polymerase coding sequence (Δ3D) which prevents its replication. Because this RNA contains the authentic 5’ and 3’ non-translated regions, and codes for all the viral proteins except the 3D polymerase, it fully recapitulates the early translation step of the replication cycle. In control cells treated with non-targeting siRNA control, the luciferase signal was steadily increasing up to four hours post-transfection of the Δ3D construct, reflecting the efficient translation of the input RNA (Fig 5D). The ILF3-90 depletion resulted in a moderate ~40% decrease in the total translation signal, while in EWSR1-depleted cells the translation of polio RNA was strongly inhibited and reached only ~16% of control (Fig 5D). These data suggest that EWSR1 may play an important role in the enterovirus IRES-mediated translation, while the ILF3-90 negative effect on enterovirus replication is likely due to its interference with the replication step of the viral life cycle.

Interestingly, while cells treated with the siRNAs against these proteins did not show any obvious signs of cytotoxicity, the viability assay based on the measurement of ATP showed significantly lower values for cells depleted of AldoA (Fig 5A and 5B, viability). We compared the ATP measurement-based viability test with an assay that detects the activity of a mitochondrial respiratory chain. In contrast to the ATP-based test, the latter assay did not detect any difference in cell viability upon depletion of any of the tested proteins (Fig 5E). This suggests that the negative effect of the AldoA depletion on enterovirus replication may result from lowered ATP level which implies a requirement for high ATP production to sustain enterovirus replication.

**Fig 6:**
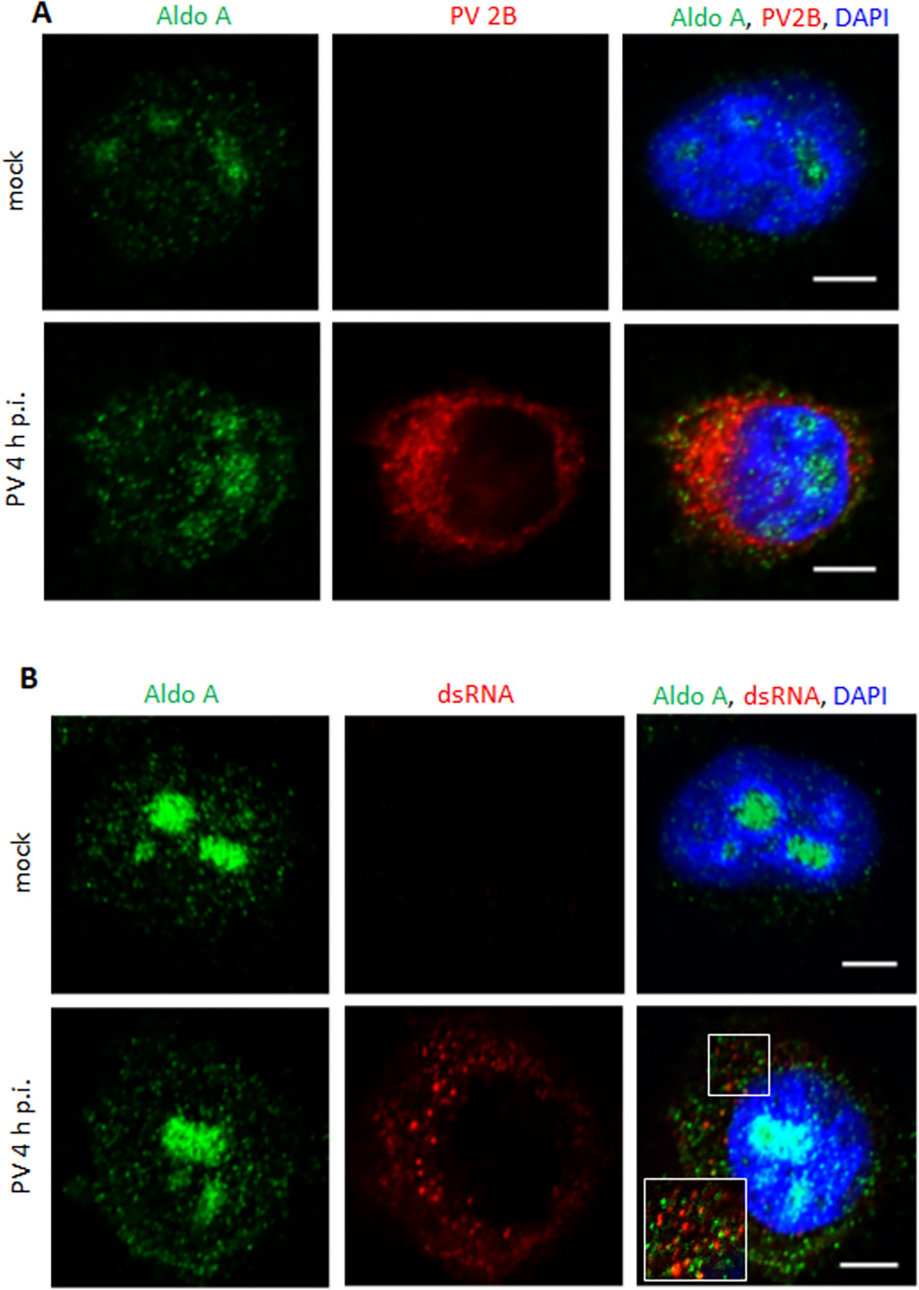
AldoA localization in infected and mock-infected cells. **A, B**. Confocal images of HeLa cells infected (or mock-infected) with 10 PFU/cell of poliovirus, fixed at 4 h p.i. and stained with antibodies against AldoA and a viral antigen 2B (A), or AldoA and dsRNA (B). Scale bar is 10 μm.

Finally, we analyzed the effect of infection on the cellular distribution of AldoA, EWSR1 and ILF3-90. In mock-infected cells, AldoA was predominantly localized in the nucleus, and in a dot-like pattern in the cytoplasm, especially in the perinuclear region, in accordance with a previous report of nuclear accumulation of AldoA in actively dividing cells [89]. In poliovirus-infected cells, the cytoplasmic AldoA signal was confined within the area of the replication organelles, as evidenced by the staining for the viral membrane-targeted protein 2B (Fig 6A). Interestingly, Aldo-A positive cytosolic punctae were often very closely associated with the signal for dsRNA, but the signals were adjacent and did not colocalize (Fig 6B).

**Fig 7:**
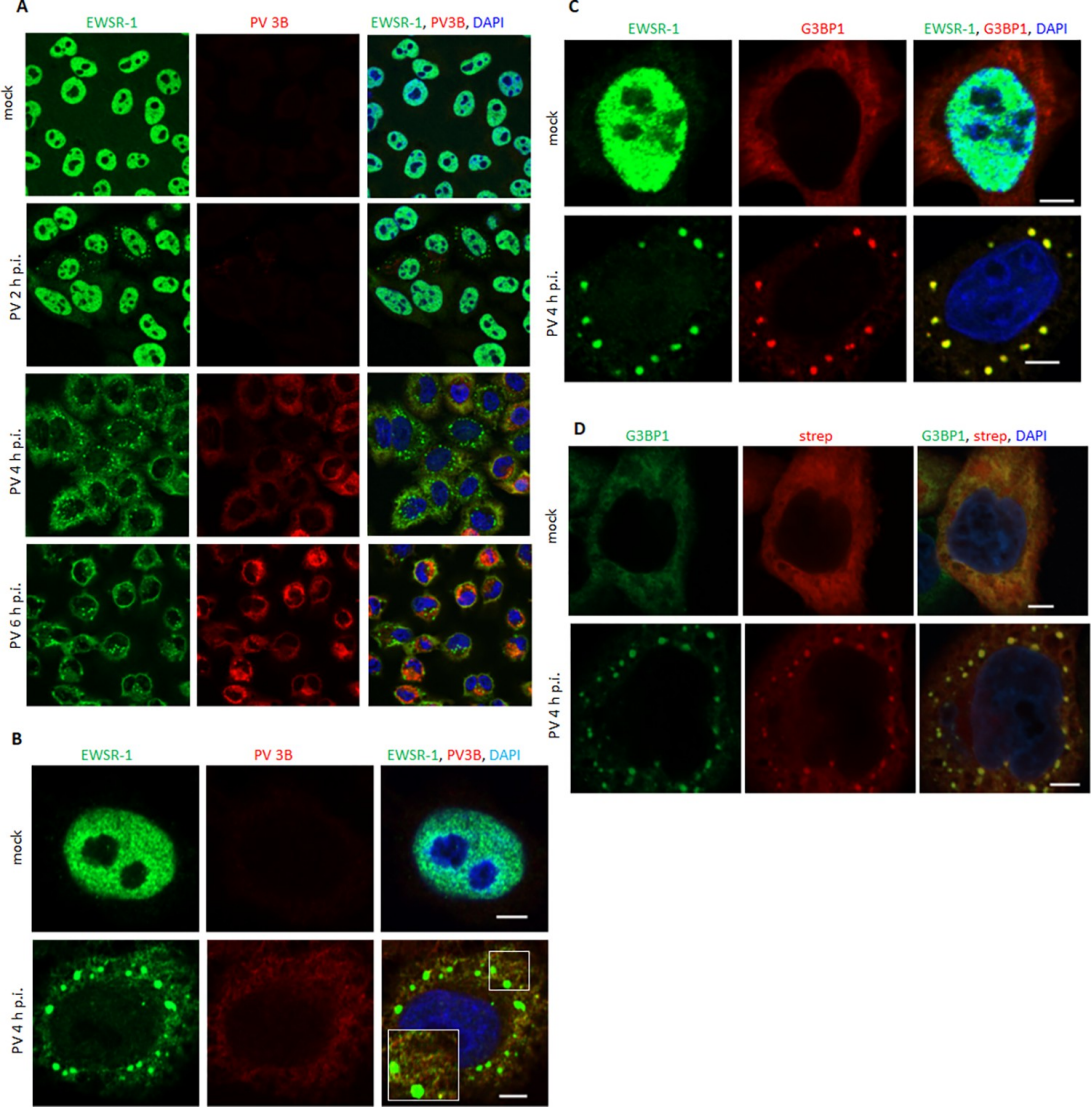
Cytoplasmic translocation of EWSR1 and its association with stress granules upon poliovirus infection. **A.** Confocal images of HeLa cells infected (or mock-infected) with 10 PFU/cell of poliovirus, fixed at 2, 4 and 6 h p.i and stained with antibodies against EWSR1 and the viral replication antigen 3B. **B.** High magnification confocal images of HeLa cells infected (or mock-infected) and processed as in A at 4 h p.i. Note the association of cytoplasmic EWSR1 signal outside of stress granules with the 3B-positive structures. **C.** Confocal images of HeLa cells infected (or mock-infected) as in A, fixed at 4 h.i. and stained with antibodies against EWSR1 and the stress granule component G3BP1. Scale bar is 10 μm. **D.** Confocal images of HeLa cells stably expressing APEX2-GARG-1060 infected (or mock-infected) as in A. C, and processed at 4 h p.i. to visualize biotinylated proteins and stained with antibodies to the stress granule component G3BP1. Scale bar is 5 μm.

EWSR1 in mock-infected cells was found exclusively in the nuclei (Fig 7A). In contrast, in poliovirus-infected cells as early as 2 h p.i. large EWSR1-positive punctae appeared in the cytoplasm, and by 4 h p.i., in the middle of poliovirus infectious cycle, all infected cells had exclusively cytoplasmic EWSR1 signal with multiple punctae. By 6 h p.i. ESWR1 signal concentrated in the perinuclear area colocalizing with the replication organelles, and the number of punctae per cell decreased, although they were still clearly detectable in the majority of the cells (Fig 7A). The non-punctate cytoplasmic EWSR1 signal in infected cells strongly colocalized with structures positive for a viral replication antigen 3B (VPg) (Fig 7B). The 3B signal may correspond to the RNA replication primer in a free form, or attached to the 5’ of viral RNAs, but may also be detected as a part of intermediate polyprotein processing products. The cytoplasmic punctae of EWSR1 in infected cells were highly reminiscent of the development of stress granules upon poliovirus infection [90–92]. To assess the relationship between EWSR1 and stress granules, we stained mock-infected and infected cells for EWSR1 and the GTPase Activating Protein (SH3 Domain) Binding Protein 1 (G3BP1), a stress granule assembly factor known to be recruited to poliovirus-induced stress granules [93]. Indeed, in infected cells, the cytoplasmic punctae of EWSR1 and G3BP1 signals colocalized perfectly, confirming that these structures are stress granules (Fig 7C). The detection of a stress granule protein upon proximity biotinylation by a GBF1-derived construct was somewhat unexpected since we are not aware of reports of GBF1 targeting to stress granules in infected or otherwise stressed cells. Thus, we analyzed the presence of biotinylated proteins in G3BP1-marked structured in APEX2-GARG-1060-expressing cells after infection. Indeed, we observed multiple bright biotinylation-positive punctae colocalizing with G3BP1-containing stress granules in the cytoplasm, indicating that components of the stress granules are labeled by the APEX2-GARG-1060 construct (Fig 7D). Whether endogenous GBF1 associates with stress granules upon infection, or this phenotype is specific to the truncated APEX2-GARG-1060 construct requires further investigation.

**Fig 8:**
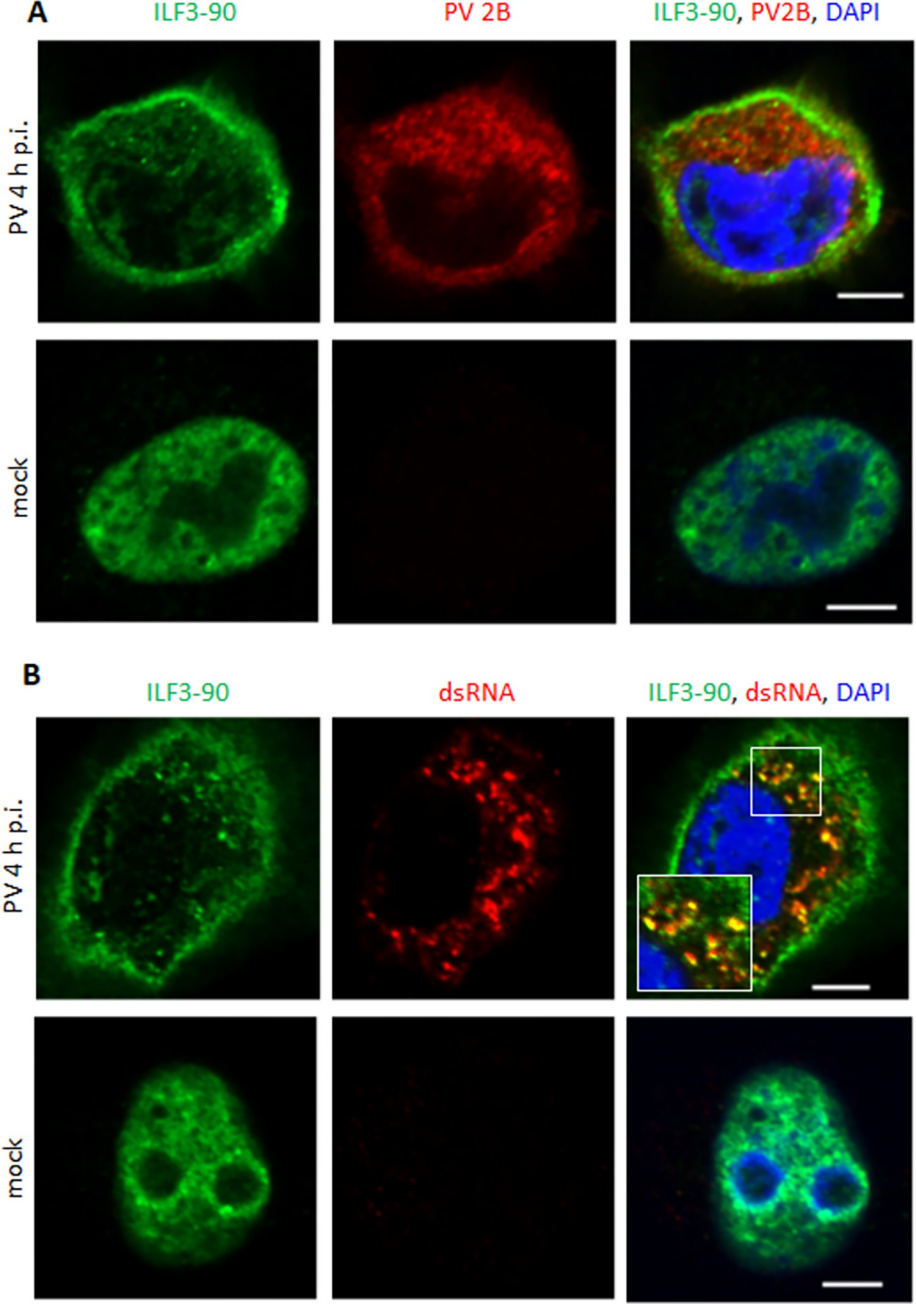
ILF3-90 associates with dsRNA in poliovirus-infected cells. **A, B.** Confocal images of HeLa cells infected (or mock-infected) with 10 PFU/cell of poliovirus, fixed at 4 h p.i. and stained with antibodies against ILF3-90 and the viral antigen 2B (**A**), or ILF3-90 and dsRNA (**B**). Scale bar is 10 μm. Note the concentration of ILF3-90 signal on the outer border of the replication organelles and its association with dsRNA inside the replication organelles.

Similar to EWSR1, ILF3-90 was localized exclusively in the nuclei of mock-infected cells (Fig 8A). Upon poliovirus infection, ILF3-90 signal became exclusively cytoplasmic and was concentrated on the outside margin of the replication organelles, with some of the ILF3-90 distributed within the inner area of the replication organelles (Fig 8A). The foci of ILF3-90 within the replication organelles strongly colocalized with the dsRNA signal, arguing that its anti-viral activity relies on its dsRNA binding capacity (Fig 8B).

Overall, our findings document novel host proteins affecting enterovirus replication and demonstrate the complex spatio-temporal dynamics of their translocation and association with the replication organelles that underscores the massive reorganization of the architecture and metabolism of enterovirus-infected cells.

## Discussion

Virtually all stages of the enterovirus replication cycle within a cell are associated with the specialized membranous structures, the replication organelles. While their morphological development is extensively documented since the early days of electron microscopy, the landscape of the host and viral proteins on the membranes of the replication organelles and their functional associations are understood only superficially. Here, we used a proximity biotinylation approach to identify proteins localized on the replication organelles in the vicinity of GBF1, a cellular factor indispensable for RNA replication of all enteroviruses tested so far, as well as some others but not all +RNA viruses [51,94–97]. Our GBF1 construct fused to the APEX2 peroxidase had two important modifications. First, the removal of the C-terminal half of GBF1 eliminated its interactions with cellular proteins non-essential for viral replication [55]. Second, our GBF1 construct had a BFA-resistant Sec7 domain, which allowed us to perform infections in the presence of BFA. Under these conditions, the endogenous GBF1 was inactivated and viral replication was supported exclusively by the APEX2-GARG-1060 construct thus increasing the specificity of the detection of proteins relevant for the functioning of the viral replication complexes.

### Our results in the context of other high throughput studies of cellular factors involved in enterovirus replication

The high throughput methods of identification of host factors involved in viral replication can be divided into two general classes—analyses based on a phenotypic response of viral replication to the depletion of a cellular factor, and unbiased methods that seek to identify all the proteins somehow associated with viral replication complexes. Each of these approaches can rely on different technical implementations which have their specific advantages and limitations. The phenotype-based methods usually use siRNA, shRNA or CRISPR-CAS9-based genetic screens targeting the expression of cellular genes, and by definition would identify functionally important host factors whose depletion either suppresses or promotes viral replication. However, these methods are likely to miss proteins important for viral replication that are also essential for cellular viability. Moreover, the prolonged depletion of a particular protein can induce unpredictable compensatory changes in the expression of other cellular factors that may complicate the interpretation of the results. The unbiased approaches, on the other hand, aim to identify all the proteins found at a specific location at a given time, but do not provide immediate information on their functional significance. The spectrum of identified proteins in unbiased approaches depends on the sample enrichment, protein labeling, purification and detection techniques, thus different proteins may be visible or hidden depending on a specific protocol. For example, in our proximity biotinylation approach, we observed that at least one protein (PI4KIIIβ) was found in the biotinylated fraction by western blot but was not identified by subsequent mass-spectrometry analysis. Similarly, while high enrichment of Arfs on the replication organelles is well documented [65,66], and Arfs are expected to be close to GBF1 on the replication organelles, we did not identify Arfs among the proteins biotinylated by our APEX2-GARG-1060 construct. This may be due to the intrinsic limitation of proximity biotinylation which depends on the accessibility of electron-rich amino-acids such as tyrosine, histidine, and tryptophan that can serve as acceptors of biotinphenoxyl radicals [98]. Thus, negative results cannot be unequivocally interpreted as the absence of the protein in the vicinity of the bait. The exposure of suitable amino-acids may be particularly limiting for the detection of small proteins like Arfs.

Both genetic screens and unbiased proteomic approaches were previously used to characterize host factors involved in enterovirus replication. RNAi screens were used by Wu et al. to identify factors important for replication of enterovirus A71 in RD cells, by Coyne et al. to find factors affecting replication of poliovirus and Coxsackievirus B3 in microvascular endothelial cells, a model of the blood-brain barrier, and by van der Sanden et al. to reveal host proteins restricting or promoting the replication of vaccine strains of poliovirus in Vero cells [99–101]. Diep et al. performed a CRISPR-CAS9 screen to identify the factors required to support the replication of rhinovirus C15 and enterovirus D68 in HeLa cells [102]. Among the most comprehensive unbiased proteomic screens, van Kuppeveld group used a quantitative characterization of the total protein abundance and phosphorylation status during the time course of Coxsackievirus B3 infection in HeLa cells [103], while Lenarcic and colleagues also used HeLa cells to analyze cellular proteins interacting with poliovirus RNA using labeling of the replicating RNA with 4-thiouracil followed by crosslinking of RNA-bound proteins [81]. Flather et al. characterized the landscape of the nuclear proteins released in the cytoplasm upon rhinovirus infection [71], and Saeed et al. defined the cellular proteins cleaved by enterovirus proteases [104]. A comparison of our dataset with those in the previous reports revealed a particularly strong overlap (as a percentage of the total number of proteins identified) with the proteins bound to poliovirus RNA [81]. From the 81 proteins reported in that study, 38 were also detected by our approach. This strongly suggests that APEX2-GARG-1060 (and by extension GBF1) localizes on the replication organelles close to the RNA replication complexes (S4 Data). Among the genetic screens, our dataset shared 14 genes with those found to be important to support enterovirus A71 replication [101], 13 genes promoting, and six genes restricting poliovirus replication from [100], as well as one gene with those supportive for the rhinovirus C15 and enterovirus D68 replication from [102]. Importantly, analysis of the proteins identified in this study revealed 112 proteins essential for cellular viability that would be difficult if not impossible to identify in genetic screens (S4 Data). Thus, it is important to use different complementary approaches to elucidate the full spectrum of host proteins involved in viral replication.

### Viral proteins

The biotinylated fraction, together with the the fully processed viral proteins, contained an increased proportion of the intermediate cleavage fragments of the poliovirus polyprotein, suggesting that APEX2-GARG-1060 on the replication membranes is localized close to the sites of active polyprotein processing. This is consistent with the requirement for GBF1 activity for the functioning of the viral RNA replication complexes and the need for the replication complex proteins to be assembled *in cis*, i.e. derived from the same polyprotein molecule [13,105–107]. On the other hand, an artificial increase in the recovery of the large polyprotein fragments due to a more efficient purification because of a higher level of biotinylated amino-acids cannot be excluded.

### Arfs and Arf effector proteins

The Arf activating function of GBF1 is essential for enterovirus replication, and all Arf isoforms massively associate with the replication organelles [55,65,66]. Thus, it is likely that in the infected cells, Arfs recruit their effector proteins to the membranes of the replication organelles which may contribute to the functioning of the replication complexes. Indeed, multiple proteins reported to interact with Arfs were biotinylated by APEX2-GARG-1060 in infected cells, with Arf1 interactors being the most numerous. Given that the depletion of Arf1 affects poliovirus replication much stronger than the depletion of any other Arf [66], further investigation of the identified Arf1 effectors may uncover novel factors important for viral replication. Among the Arf effector proteins we identified in our dataset, Cytohesin2/ARNO, a BFA-insensitive ArfGEF is of particular interest. ARNO in non-infected cells regulates endosomal membrane trafficking and actin polymerization at the plasma membrane through activation of Arf6, but it can also activate other Arfs, and is itself an Arf1 and Arf6 effector [60,108,109]. Thus, it is likely that in addition to GBF1-mediated Arf activation, ARNO-dependent Arf activation may operate on the replication organelles. The existence of such a pathway may contribute to the establishment of resistance to inhibitors of GBF1, such as BFA and similar molecules.

### Both replication-promoting and replication-restricting cellular factors are identified by GBF1-specific biotinylation

We analyzed the effect of depletion of several proteins belonging to different functional groups that were significantly enriched in the dataset from the infected cells on viral replication. AldoA was one of several glycolytic enzymes biotinylated by APEX2-GARG-1060 in poliovirus-infected cells, and its depletion significantly inhibited viral replication. The infection-specific biotinylation of multiple glycolytic enzymes suggests an active supply of the replication organelles with glycolysis pathway-derived metabolites. Recently, the recruitment of glycolytic enzymes to replication organelles of tombusviruses, a group of positive-strand plant viruses has been discovered, and it was shown that these enzymes participate in generating a high local level of ATP required to support viral replication [110–112]. AldoA-dependent ATP production was shown to be important for the replication of the Japanese encephalitis virus, a positive-strand RNA virus of the *Flaviviridae* family [113]. The importance of *de novo* nucleotide synthesis on picornavirus replication organelles is highlighted by the previous observations that partially purified membrane-associated replication complexes more efficiently incorporate in the replicating RNA exogenously added nucleoside mono and diphosphates than nucleoside triphosphates [114]. It is likely that the recruitment to replication organelles of glycolytic enzymes that may support the local nucleotide synthesis is a conserved feature of replication of positive-strand RNA viruses.

Among the highly-enriched RNA-binding proteins, we focused on EWSR1 and ILF3 whose depletion consistently affected the virus replication. EWSR1 is an RNA and DNA binding multifunctional protein involved in different networks of regulation of gene expression [115]. The ILF3 gene is expressed as two major isoforms of 110 and 90 KDa that regulate multiple steps of RNA metabolism in the nucleus and the cytoplasm [116]. Depletion of ILF3-90 but not IF3-110K stimulated the infectious virus yield of poliovirus and Coxsackievirus B3. Thus ILF3-90K may be a broad anti-enterovirus factor. Our data contrast with the previously reported virus-specific inhibitory activity of ILF-3 depending on its binding to rhinovirus but not poliovirus IRES [88]. This may reflect the difference in the experimental systems used, but the dsRNA-binding capacity of ILF3-90 supported by our data likely underlies its broad anti-viral effect observed in our study.

The depletion of ESWR1 significantly inhibited the translation of replication-deficient poliovirus RNA, suggesting that this protein may be involved in IRES-dependent translation initiation. The effect of ILF3 depletion on translation was much smaller, arguing that it mostly affects the replication step of the viral life cycle. The specific mechanisms by which ESWR1 and ILF3 modulate the translation, replication and, probably, encapsidation, of the enterovirus RNAs require further investigation.

Both EWSR1 and ILF3-90 were strictly confined to the nuclei before infection, while in infected cells starting from the middle of the infectious cycle their localization was exclusively cytoplasmic. The disruption of the nucleo-cytoplasmic barrier is caused by the cleavage of nucleoporins by enterovirus protease 2A, which also cleaves a translation initiation factor eIF4G. This results in the rapid inhibition of both mRNA export from the nucleus and cap-dependent translation of cellular mRNAs [18–20]. In addition, the viral proteases 3C and 3CD cleave the core components of RNA polymerase II [117,118]. The rapid and profound inactivation of cellular gene transcription and translation implies that the proteins present in the cell before the infection must contain the full complement of factors required to support viral replication. This also implies that the cells must have in place some anti-viral measures ready to be deployed without significant input from new gene expression. The nuclear depot of RNA-binding proteins thus may represent an important resource of both pro- and anti-viral factors. Interestingly, the major ILF3-90 signal in infected cells was outlining the outer border of the replication organelle area. It is tempting to speculate that this represents the visual manifestation of the protective function of the membranous scaffold of the replication organelles hiding the active replication complexes from the access of anti-viral factors.

In the initial screen, we relied on previously characterized siRNAs targeting the expression of cellular proteins, but we did not test their efficacy in our cells. It may be that some moderate or inconsistent effects observed in the replicon replication and infection assays were due to the limited efficacy of the chosen siRNAs in our system. It also should be noticed that the protein abundance upon the proximity biotinylation-based detection reflects the combination of three variables- the concentration of the protein in a cell, its retention close to the bait, and the exposure of the amino-acids that can accept the biotinphenoxyl radicals. This may skew the representation of the actual composition of proteins at the bait location, thus the less abundant proteins specifically detected at the replication organelles should also be investigated in the future for their role in the enterovirus infection.

Overall, our findings significantly increase the knowledge of cellular proteins associated with enterovirus replication organelles and provide an important resource for the rational approach for the development of antiviral strategies targeting factors supporting replication of diverse enteroviruses.

## Materials and methods

### Cells and viruses

HeLa and RD cells were maintained in DMEM high glucose modification supplemented with pyruvate and 10% FBS. Retrovirus packaging cell line GP2-293 was maintained in DMEM high glucose modification supplemented with 10% FBS. Cell viability was determined using either CellTiter-Glo kit (Promega) or XTT assay (Thermo Fisher) that detect the level of cellular ATP, or the activity of the mitochondrial respiratory chain enzymes, respectively, according to the manufacturers’ recommendations. Poliovirus type I (Mahoney) and Coxsackie virus B3 (Nancy) were rescued using the plasmids pXpA and p53CB3/T7 coding for the full-length viral cDNAs under the control of a T7 promotor kindly provided by Dr. Raul Andino (University of California, San Francisco) and Prof. van Kuppeveld (University of Utrecht, the Netherlands), respectively. The viruses were propagated in HeLa cells, viral titers were determined by plaque or TCID_50_ assays on RD cells grown on 6- or 96-well plates, respectively, using 10x dilutions of the virus preparations. TCID_50_ titers were calculated using Kärber’s formula [119].

### Plasmids

APEX2 coding sequence [57] with the FLAG epitope at the N-terminus optimized for expression in human cells was synthesized by Invitrogen (GeneArt service). For the transient expression, the FLAG-APEX2 was fused in-frame upstream of the GBF1 truncated after HDS1 domain and containing a BFA-resistant Sec7 domain from ARNO (GARG-1060) in a mammalian expression vector pCI (Promega) generating a plasmid pCI-FLAG-APEX2-GARG-1060. For stable expression, the FLAG-APEX2-GARG-1060 insert was cloned into the retroviral vector plasmid pLNCX2 (Takara Bio). Cloning details are available upon request. Plasmid pEGFP-GARG-1060 coding for the truncated GBF1 with a BFA-resistant Sec7 domain under the control of a CMV promotor was described in [55]. Plasmids pXpA-RenR and pRib-CB3-RLUC coding for cDNAs of polio and Coxsackie B3 replicons with Renilla luciferase substituting the capsid coding region P1 under the control of a T7 promotor were described in [97,120]. Plasmid pXpA-RenRΔ3D lacks a fragment of 460 nt between two HindIII sites in the coding region of poliovirus 3D polymerase and thereby encodes a replication-defficient translation-competent polio replicon RNA.

### Establishment of a HeLa cell line stably expressing APEX2-GBF1 construct

Retrovirus packaging cell line GP2-293 (Takara Bio) expressing Moloney murine leukemia virus gag and pol genes was co-transfected with pLNCX2 vector with the FLAG-APEX2-GARG1060 insert and a plasmid coding for the vesicular stomatitis virus envelope glycoprotein (Takara Bio) using Mirus 2020 DNA transfection reagent (Mirus). The infectious virions were harvested in the culture supernatant 48 h post-transfection. HeLa cells seeded into a 6-well plate were transduced with the freshly produced retrovirus virions in the complete growth medium supplemented with 10 μg/ml Polybrene (Millipore Sigma). The plate was centrifuged at 1,200g for 1 h at 32°C to enhance the transduction efficiency and kept at 37° C for 18 h. The next day, the transduction medium was replaced with a fresh complete growth medium, and cells were incubated overnight. Forty-eight hours after the start of transduction, cells were transferred into a T-25 flask, and the drug-resistant colonies were selected with 300 μg/ml G418 (VWR Life Science) for two weeks. The resistant colonies were pooled, and the stable cell lines were maintained in the complete growth medium supplemented with 300 μg/ml G418. At this point, approximately 60% of the cells showed the expression of the transgene. For a clonal selection, the cells were seeded at a density of ~0.3 cell/well in a 96 well plate and the colonies established from the individual cells were screened for the transgene expression. A colony that showed >90% uniform pattern of a functional transgene expression as demonstrated by anti-FLAG staining, biotinylation reaction, and polio replicon replication in the presence of 2μg/ml of brefeldin A (Millipore Sigma) was expanded and used for the rest of the study.

### Antibodies

#### Cellular proteins

Mouse monoclonal antibodies: anti-GBF1 (BD Biosciences (612116)), anti-EWSR1 (Novus Biologicals (NBP1-92686)), anti-ACBD3 (Millipore Sigma (SAB1405255)), anti-β-actin antibodies conjugated with horseradish peroxidase (HRP) (Millipore Sigma (A3854)). Rabbit polyclonal antibodies: anti-OSBP (ProteinTech (11096–1)), anti-AldoA (Millipore Sigma (HPA004177)), anti-PI4KIIIβ (Millipore Sigma (06–578)), anti-ILF3-90 (Millipore Sigma (HPA001897)), anti-FLAG tag (Thermo Fisher (PA1-984B)). Rabbit monoclanl anti-G3BP1 antibody (Cell Signaling (61559T)).

#### Viral antigens

Mouse monoclonal anti-poliovirus VP3, 2B, 2C, and 3A, and rabbit polyclonal antibodies against poliovirus 3B were a gift from prof. Kurt Bienz (University of Bazel, Switzerland) and were partially described in [121–123]. Rabbit polyclonal anti-poliovirus 3D antibodies were custom generated by Chemicon and were described in [36]. Mouse monoclonal anti-dsRNA antibody J2 was from English & Scientific Consulting Kft.

Secondary Alexa dye fluorescent antibody and streptavidin conjugates were from Thermo Fisher, HRP secondary antibody conjugates were from Amersham or KPL. Streptavidin HRP conjugate was from Millipore Sigma (RPN1231).

### Biotinylation reaction

Depending on the future analysis, HeLa cells stably expressing FLAG-APEX2-GARG1060 were seeded in either a 12-well plate with or without coverslips, a T-25, or a T-225 flask. The cells were infected (or mock-infected) with 10 PFU/cell of poliovirus, and incubated in the presence of 2 μg/ml BFA. At 30 min before the indicated times post-infection, the incubation medium was replaced with pre-warmed DMEM with 500 μM biotinyl tyramide (biotin-phenol) (Chemodex). For the biotinylation reaction, the medium was replaced with PBS containing 20 mM H_2_O_2_ (Millipore Sigma) and incubated for three min at 37°C. Then the cells were washed three times with PBS and either immediately fixed with 4% formaldehyde (Electron Microscopy Sciences) in PBS for microscopy, or lysed in RIPA lysis buffer supplemented with a proteinase inhibitor cocktail (Millipore Sigma) followed by sonication. The sonicated RIPA lysates were used either directly for SDS-PAGE and western blotting, or for further purification of biotinylated proteins.

### Purification of biotinylated proteins

The whole-cell lysates were mixed with the streptavidin magnetic beads (Pierce) equilibrated in RIPA buffer and incubated on a rotator for 1 h at room temperature. The beads were collected using a magnetic rack and washed three times with RIPA buffer. The bound proteins were eluted by boiling the beads in 40 μL of 3X Laemmli sample buffer supplemented with 2 mM biotin and 20 mM dithiothreitol for 10 min. The beads were removed using a magnetic rack, and the eluates were kept at -80°C for further analysis.

### siRNAs

The following sense siRNA sequences targeting the expression of human genes were used:

**Table:** 

AldoA	5′-CCGAGAACACCGAGGAGAA-3′	[124]
EWSR1	5’-CUACUAGAUGCAGAGACCC-3’	[125]
HNRNPA0	5’-CAGACCAAGCGCUCCCGUU-3′	[126]
HNRNPH2	5’-CAUGAGAGUACAUAUUGAA-3′	[127]
HNRNPH3	5’-GACAGUACGACUUCGUGGA-3′	[126]
HNRNPR	5’-GGAGUAUGGAGUAUGCUGU-3′	[126]
HNRNPU	5’-AAAGACCACGAGAAGAUCAUG-3’	[128]
ILF3-110	5’-GCGGAUCCGACUACAACUACG-3′	[129]
ILF3-90	5’-CUUCCUAGAGCGUCUAAAAGU-3′	[129]
KHDRBS1	5’-GGACCACAAGGGAAUACAA-3′	[130]
LDHA	5′-AAGACAUCAUCCUUUAUUCCG-3′	[131]
LDHB	5’-GUACAGUCCUGAUUGCAUC-3′	[132]
PKM	5′-CCACGAGCCACCAUGAUCC-3′	[124]
RBMX	5’-CGGAUAUGGUGGAAGUCGA-3’	[133]
SYNCRIP	5’-GCUAGUUGCACAUAGUGAU-3′	[126]

The siRNA duplexes were synthesized with 3’ UU overhangs by Dharmacon. The siRNAs were transfected into HeLa cells using Dharmafect 1 transfection reagent according to the manufacturer’s protocol, and the experiments were performed ~72h after siRNA transfection. As a non-targeting control siControl#1 (Ambion) was used.

### Microscopy

Cells grown on a coverslip in a 12-well plate were fixed with 4% formaldehyde (Electron Microscopy Sciences) in PBS for 20 min, washed three times with PBS and permeabilized for 5 min in 0.2% Triton-X100 (Millipore Sigma). The cells were incubated sequentially with the primary and secondary antibodies diluted in PBS with 3% blocking reagent (Amersham) for 1 h each with 3x PBS washes in between. Confocal images were taken with Zeiss LSM 510 microscope under the control of ZEN software (Zeiss). All images from one experiment were taken under the same microscope settings. Structural illuminated microscopy super-resolution (SIM) images were taken with Nikon A1R microscope under the control of NIS-Elements software (Nikon). Digital images were processed with Adobe Photoshop software for illustrations, all changes were applied to the whole image, and images from the same experiments were processed with the same settings.

### Replicon assay

Replicon assays were performed essentially as described in [120]. Briefly, purified replicon RNA was transfected into HeLa cells grown on 96 well plates using TransIT mRNA transfection reagent (Mirus), and the cells were incubated in the growth medium supplemented with 5μM of cell-permeable Renilla luciferase substrate EnduRen (Promega) at 37°C directly in an ID3 multiwell plate reader (Molecular Devices). The measurements were taken every hour for 18 hours, the data were processed using GraphPad Prism software. Translation assay with a replicon RNA with the Δ3D mutation was perfomed similarly, but with 5x more RNA used for transfection. Total replication or translation signals were calculated as the area under the curve for the kinetic luciferase measurement for each well, the signal from at least 16-wells was averaged for each sample, unpaired t-test was used to compare the differences within pairs of experimental and control conditions, p<0.05 was considered significant.

### Proteomics analysis

Biotinylated proteins collected from infected (or mock-infected) HeLa cells grown on a 225cm^2^ flask (approximately 3E7 cells/flask) from five independent experiments were pooled together and run into 12% SDS-PAGE for ~1cm. The gel was stained with Coomassie blue and the gel slices containing proteins from infected and mock-infected cells were excised and processed for proteomics analysis at the Harvard proteomics facility as follows:

#### Sample Preparation procedure

Gel slices were washed in 50% acetonitrile and rehydrated with 50 mM ammonia bicarbonate trypsin solution for overnight digestion at 37°C. The next day peptides were extracted with a series of elution and completely dried down in a speed vac. The samples were solubilized in 0.1% formic acid in water for analysis by tandem mass spectrometry.

#### Mass spectrometry analysis

The LC-MS/MS experiment was performed on a Lumos Tribrid Orbitrap Mass Spectrometer (Thermo Fischer) equipped with Ultimate 3000 (Thermo Fisher) nano-HPLC. Peptides were separated onto a 150μm inner diameter microcapillary trapping column packed first with approximately 2cm of C18 Reprosil resin (5μm, 100 Å, Dr. Maisch GmbH, Germany) followed by PharmaFluidics (Gent, Belgium) 50cm analytical column. Separation was achieved by applying a gradient from 5–27% acetonitrile in 0.1% formic acid over 90 min at 200 nl/min. Electrospray ionization was enabled by applying a voltage of 2 kV using a homemade electrode junction at the end of the microcapillary column and sprayed from metal tips (PepSep, Denmark). The mass spectrometry survey scan was performed in the Orbitrap in the range of 400–1,800 m/z at a resolution of 6×10^4^, followed by the selection of the twenty most intense ions (TOP20) for CID-MS2 fragmentation in the Ion trap using a precursor isolation width window of 2 m/z, AGC setting of 10,000, and a maximum ion accumulation of 100 ms. Singly charged ion species were not subjected to CID fragmentation. The normalized collision energy was set to 35 V and an activation time of 10 ms. Ions in a 10 ppm m/z window around ions selected for MS2 were excluded from further selection for fragmentation for 60s.

#### Data analysis

Raw data were submitted for analysis in Proteome Discoverer 2.4 (Thermo Scientific) software. Assignment of MS/MS spectra was performed using the Sequest HT algorithm by searching the data against a protein sequence database including all entries from our Uniport_Human2018_SPonly database as well as other known contaminants such as human keratins and common lab contaminants. Quantitative analysis between samples was performed by label-free quantitation (LFQ). Sequest HT searches were performed using a 10 ppm precursor ion tolerance and requiring N-/C termini of each peptide to adhere to trypsin protease specificity while allowing up to two missed cleavages. Methionine oxidation (+15.99492 Da), as well as deamidation (+ 0.98402 Da) of asparagine and glutamine amino acids, were set as variable modifications. Special modification of 1xbiotin-tyramide (+361.14601 Da) on tyrosine amino acid residues was used as a variable modification. All cysteines were set to a permanent modification with carbamidomethyl (+ 57.02146 Da) due to an alkylation procedure. All MS2 spectra assignment false discovery rate (FDR) of 1% on both protein and peptide levels was achieved by applying the target-decoy database search by Percolator [134].

#### Gene ontology analysis

The sets of proteins identified in the infected and mock-infected samples were analyzed for Gene Ontology (GO) term enrichment using PANTHER classification system web tool [69] against all *Homo sapiens* protein-coding genes using Fisher’s exact test and Bonferroni correction for multiple testing. Only the statistically significant enrichment results with p<0.05 are reported.

#### Factors essential for cellular viability

The genes coding for the proteins identified in the infected sample were analysed against the essential genes list using the data of the Ahilles genome-scale CRISPR screens in cancer cell lines on DepMap portal [135,136].

## Supporting information

S1 Data Dataset from LFQ proteomic analysis of the biotinylated proteins purified from HeLa cells stably expressing APEX2-GARG-1060 infected, or mock-infected with 10 PFU/cell of poliovirus for 6 h before the biotinylation reaction. (XLSX)

S2 Data PANTHER Gene Ontology (GO) Overrepresentation Test (Release 20210224) with the proteomic datasets from the poliovirus-infected and mock-infected cells (S1 Data). GO Ontology database DOI: 10.5281/zenodo.5228828 Released 2021-08-18. Analysis performed: Fisher exact test with Bonferroni correction. Only statistically significant enrichments (p-value <0.05) are shown.
(XLSX)

S3 Data Analysis of potential interactors from the proteomics datasets from the poliovirus-infected and mock-infected cells (S1 Data) using the Biogrid database of curated interaction data [67,68]. (XLSX)

S4 Data Analysis of literature on the association of the proteins from the proteomics datasets from the poliovirus-infected and mock-infected cells (S1 Data) for their involvement in enterovirus replication, and identification of the genes essential for the cellular viability. (XLSX)

S1 Fig HeLa cells were transfected with siRNAs specific to the indicated cellular proteins, or non-targeting control siRNA. Polio replicon replication and poliovirus infection assays were performed 72 h post siRNA transfection The total replication signal was calculated as the area under the corresponding kinetics curves. For the infection assay, HeLa cells were infected with an MOI of 10 of poliovirus (or mock-infected) The cells were lysed at 4 h p.i and processed for western blot with anti-poliovirus 2C antibodies. Underlined proteins were taken for further analysis. KHDRBS1 depletion is a positive control for a cellular factor known to affect poliovirus replication [70]. Each assay was performed at least twice for each protein, representative results are shown.
(PDF)
