# Supplementary figures and images for: A Proximity biotinylation assay with a host protein bait reveals multiple factors modulating enterovirus replication
Source: PLoS Pathog. 2022 Oct 28;18(10):e1010906. doi: 10.1371/journal.ppat.1010906 (PMC9645661; doi:10.1371/journal.ppat.1010906)

replicon assay

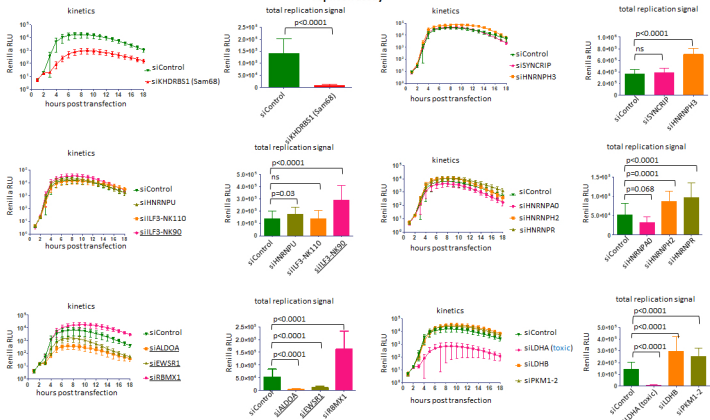

**infection assay**

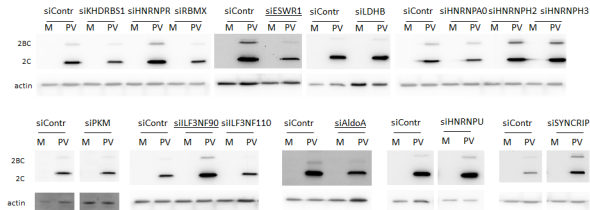

Supplementary Figure 1

Supplement: S1 Fig — Polio replicon replication and poliovirus infection assays were performed 72 h post siRNA transfection The total replication signal was calculated as the area under the corresponding kinetics curves. For the infection assay, HeLa cells were infected with an MOI of 10 of poliovirus (or mock-infected) The cells were lysed at 4 h p.i and processed for western blot with anti-poliovirus 2C antibodies. Underlined proteins were taken for further analysis. KHDRBS1 depletion is a positive control for a cellular factor known to affect poliovirus replication [70]. Each assay was performed at least twice for each protein, representative results are shown. (PDF) [file ppat.1010906.s005.pdf]
